# The *Ruegeria pomeroyi acuI* Gene Has a Role in DMSP Catabolism and Resembles *yhdH* of *E. coli* and Other Bacteria in Conferring Resistance to Acrylate

**DOI:** 10.1371/journal.pone.0035947

**Published:** 2012-04-26

**Authors:** Jonathan D. Todd, Andrew R. J. Curson, Matthew J. Sullivan, Mark Kirkwood, Andrew W. B. Johnston

**Affiliations:** School of Biological Sciences, University of East Anglia, Norwich, United Kingdom; University of Delhi, India

## Abstract

The *Escherichia coli* YhdH polypeptide is in the MDR012 sub-group of medium chain reductase/dehydrogenases, but its biological function was unknown and no phenotypes of YhdH^−^ mutants had been described. We found that an *E. coli* strain with an insertional mutation in *yhdH* was hyper-sensitive to inhibitory effects of acrylate, and, to a lesser extent, to those of 3-hydroxypropionate. Close homologues of YhdH occur in many Bacterial taxa and at least two animals. The acrylate sensitivity of YhdH^−^ mutants was corrected by the corresponding, cloned homologues from several bacteria. One such homologue is *acuI*, which has a role in acrylate degradation in marine bacteria that catabolise dimethylsulfoniopropionate (DMSP) an abundant anti-stress compound made by marine phytoplankton. The *acuI* genes of such bacteria are often linked to *ddd* genes that encode enzymes that cleave DMSP into acrylate plus dimethyl sulfide (DMS), even though these are in different polypeptide families, in unrelated bacteria. Furthermore, most strains of Roseobacters, a clade of abundant marine bacteria, cleave DMSP into acrylate plus DMS, and can also demethylate it, using DMSP demethylase. In most Roseobacters, the corresponding gene, *dmdA*, lies immediately upstream of *acuI* and in the model Roseobacter strain *Ruegeria pomeroyi* DSS-3, *dmdA-acuI* were co-regulated in response to the co-inducer, acrylate. These observations, together with findings by others that AcuI has acryloyl-CoA reductase activity, lead us to suggest that YdhH/AcuI enzymes protect cells against damaging effects of intracellular acryloyl-CoA, formed endogenously, and/or via catabolising exogenous acrylate. To provide “added protection” for bacteria that form acrylate from DMSP, *acuI* was recruited into clusters of genes involved in this conversion and, in the case of *acuI* and *dmdA* in the Roseobacters, their co-expression may underpin an interaction between the two routes of DMSP catabolism, whereby the acrylate product of DMSP lyases is a co-inducer for the demethylation pathway.

## Introduction

The compatible solute dimethylsulfoniopropionate (DMSP) is made by many marine phytoplankton, including dinoflagellates, diatoms, and coccolithophores, several marine macroalgal seaweeds and a few land plants [1]. This zwitterion probably acts as an osmolyte, although other anti-stress functions have been suggested [2]. Its high concentrations in the producing organisms (∼0.5 M in some cases) coupled to their wide distributions in the oceans, means that DMSP is one of the most abundant (∼10^9^ tons made *per annum*) organic, sulfur-containing molecules on Earth.

Furthermore, the DMSP that is released following the death or damage of the producing organisms provides an important nutrient source for many marine microbes, including the prodigiously abundant SAR11 clade, and it is therefore a key component of the global sulfur cycle [3], [4].

The catabolic fate of DMSP is complex, and varied. Some of the eukaryotic DMSP producers can themselves cleave it into acrylate plus the volatile dimethyl sulfide (DMS), as can many marine bacteria, and a few fungi (see [5]). Although the enzyme activity for this reaction is generically termed “DMSP lyase”, biochemical and genetic studies show that widely divergent enzymes, which occur in different microbes, can cleave DMSP into these products. To date, no less than five “Ddd” (DMSP-dependent DMS) gene products (DddL, DddP, DddQ, DddW and DddY) have been found in different marine bacteria, and although they all catalyse the same cleavage of DMSP into acrylate plus DMS, they are in different polypeptide families [6], [7]. A sixth bacterial “Ddd” enzyme, termed DddD, also generates DMS by cleaving DMSP, but in this case, the resultant C3 compound is 3-hydroxypropionate (3HP) [8].

In addition to these cleavage pathways, DMSP can also be catabolised in a completely different manner, in which the initial step involves demethylation to methylmercaptopropionate (MMPA), which is then further catabolised to methane thiol and acetate [9], [10]. The gene responsible for the initial demethylation is termed *dmdA*, which occurs in two groups of abundant marine α-Proteobacteria, namely a lineage known as the Roseobacters and also in the hugely populous SAR11 clade; thus, demethylation accounts for most of the global DMSP catabolic flux, although it does not liberate any DMS (see [7]).

**Figure 1:**
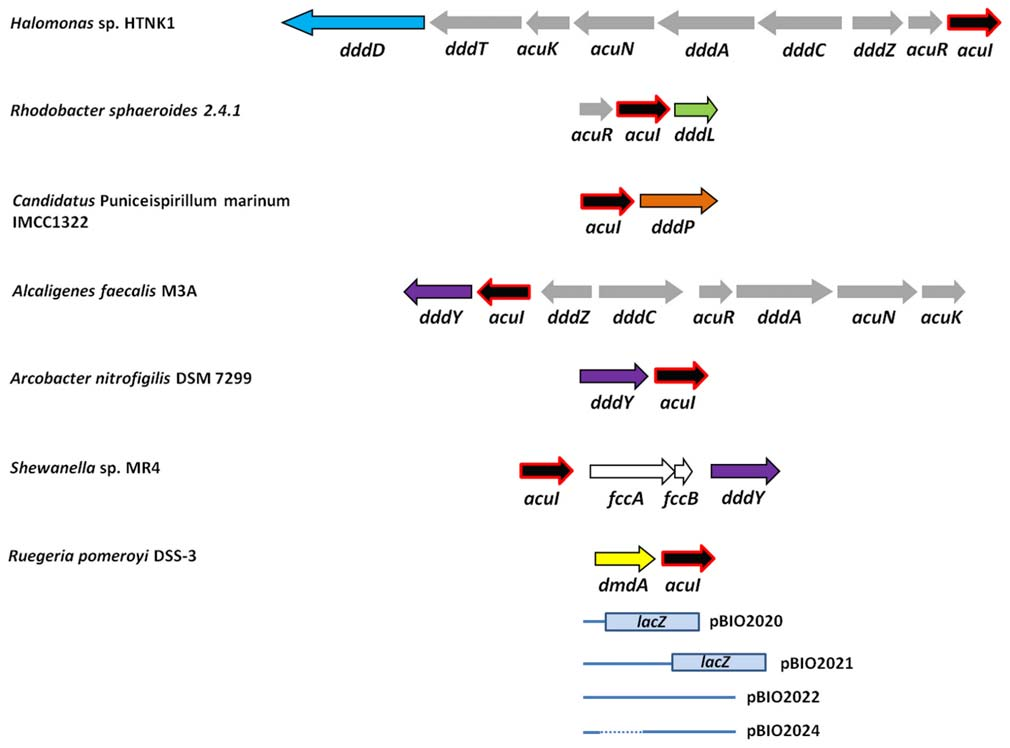
Locations of *acuI* relative to various *ddd* and *dmdA* genes in different bacteria. Locations of the *acuI* genes relative to those that encode DMSP demethylase (*dmdA* – yellow fill) or the *ddd* genes that encode the DddD, DddL, DddP and DddY DMSP lyases (arrows filled with various colors) are shown. Grey-filled arrows signify genes with other, known roles in DMSP catabolism. The *fccA* and *fccB* genes in *Shewanella* sp. MR4 encode a flavocytochrome *c* and a tetraheme cytochrome *c* respectively. Gene tags from left to right as shown above are: *Halomonas* HTNK1: ACV84065 to ACV84073 inclusive *Rhodobacter sphaeroides* 2.4.1: RSP_1433 to RSP_1435 inclusive *Canididatus* Puniceispirillum marinum IMCC1322: SAR116_1428, SAR116_1427 *Alcaligenes faecalis* M3A: ADT64689 to ADT64696 inclusive *Arcobacter nitrofigilis* DSM 7299: Arnit_0113, Arnit_0112 *Shewanella* sp. MR4: Shewmr4_2154 to Shewmr4_2151 inclusive *Ruegeria pomeroyi* DSS-3: SPO1913, SPO1914 Also shown are the dimensions of the *Ruegeria pomeroyi* DSS-3 *dmdA-lacZ* and *acuI-lacZ* fusion plasmids (pBIO2020 and pBIO2021 respectively) in which the reporter *lacZ* gene in pBIO1878. The cloned *R. pomeroyi* DNA (shown as blue lines) was cloned into pBIO1878 to form pBIO2022 (*acuI+dmdA*) and pBIO2024 (*dmdA* alone) for the complementation tests.

In several bacteria, the “primary” *ddd* genes, whose lyase products act directly on DMSP, are in clusters, together with other genes that are variously involved in the import of DMSP, in downstream catabolic steps that feed into central metabolism, or in their transcriptional regulation in response to the appropriate co-inducer molecule (see Figure 1 for some examples of these).

In addition, several different *ddd* gene clusters, in a range of bacteria, contain another gene, termed *acuI* (acrylate utilisation). First described in the α-Proteobacterium, *Rhodobacter sphaeroides* strain 2.4.1 [11], *acuI* is the central gene of a three-gene operon whose promoter-proximal *acuR* encodes a regulator in the TetR family, and whose promoter-distal gene, *dddL*, encodes a DMSP lyase that cleaves DMSP into acrylate plus DMS ([12], Figure 1). Although *R. sphaeroides* does not grow well on acrylate as sole carbon source, it can catabolise ^14^C-labelled acrylate substrate, with the concomitant release of labelled ^14^CO_2_. An AcuI^−^ mutant of *R. sphaeroides* was less effective in this transformation, and, strikingly, it was hypersensitive to the inhibitory effects of acrylate compared to the wild type [11]. Significantly, *acuI* is not only linked to other *ddd* genes that encode different types of DMSP lyases, but is also next to the DMSP demethylation gene *dmdA* in the model Roseobacter strain, *Ruegeria pomeroyi* [6], [13].

AcuI is in the “medium chain reductase/dehydrogenase” (MDR) family, members of which are widespread and occur in all Domains of life. In a detailed sequence analysis, Hedlund *et al.* [14] delineated 86 different MDR sub-families, with functions ranging from quinone reductases to a ζ-crystallin in vertebrate lens. According to this scheme, AcuI polypeptides are in the MDR012 sub-family, which is also termed the YhdH group, in recognition of the *Escherichia coli* gene product of that name, which is 54% identical to AcuI of *Rhodobacter*. The function of *E. coli* YhdH is unknown and no mutant phenotypes have been reported. Although the overall fold structure of the YhdH polypeptide resembles that of quinone reductases, it has very little sequence similarity to any known enzymes that act on quinones [15]. Furthermore, the YhdH polypeptide lacks a Zn co-factor, which distinguishes it from the many zinc-containing alcohol dehydrogenases and several other members of the MDR super-family [14], [15], [16]. Thus, AcuI is not a “putative Zn-dependent oxidoreductase”, as suggested by its annotation (http://www.ncbi.nlm.nih.gov/protein/ABA77575.1).

Recent biochemical evidence that complements these genetic links between AcuI and acrylate has recently shown that AcuI of *Rhodobacter sphaeroides* has acryloyl-CoA reductase activity, catalysing the conversion of acryloyl-CoA to propionyl-CoA [13]. Here, we present more insights into the function and distribution of *acuI*-like genes and their products in a wide range of bacteria, including those that do and those that do not catabolise DMSP. We present a model that indicates that AcuI-like enzymes have an unusual and widespread general role in bacteria, as well as affecting how DMSP can be catabolised.

## Results

### *acuI*-like genes are near some, but not all, the *ddd* and *dmdA* genes involved in DMSP catabolism

It had been noted that there were *acuI*-like genes near some bacterial *ddd* and *dmdA* genes involved in DMSP catabolism. In a more thorough examination of these linkage relationships, we found that the distributions of *acuI* genes in relation to each of the individual types of *dmdA* and *ddd* genes in different bacteria are instructive, as follows.

Of the seven known enzymes that act directly on DMSP, either demethylating it (DmdA) or cleaving it to release DMS (DddD, DddL, DddP, DddQ, DddW and DddY), all but two of the corresponding genes (*dddQ* and *dddW*) are closely linked to *acuI* in at least one bacterial strain. These are considered individually in the following section.

#### *dmdA*

The *dmdA* gene is largely confined to two groups of marine bacteria (see [6], [7]). One of these, the Roseobacters, comprises several genera in a sub-group of the Rhodobacterales. These are abundant and widespread in the oceans and coastal waters and most of them can catabolise DMSP – indeed, several individual strains can both demethylate it and cleave it, releasing DMS [17]. This is due to their possession of both the DmdA demethylase plus one or more DMSP lyases (DddD, DddL, DddP, DddQ and/or DddW), each of which generates acrylate or 3HP plus DMS. Individual Roseobacter strains have various portfolios of these different lyases [6], [18].

**Figure 2:**
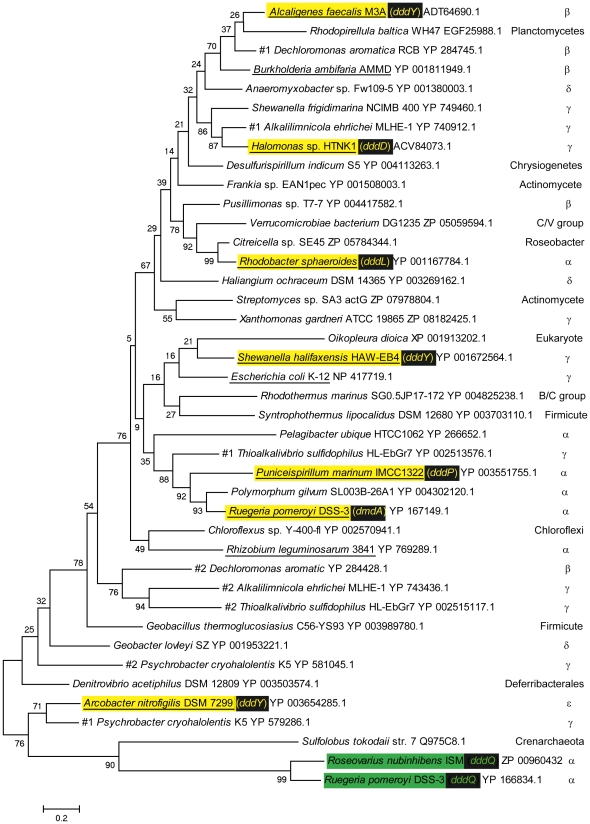
Molecular phylogenetic analysis of selected AcuI polypeptides. Strains, clades, and protein identification numbers of a selection of the AcuI polypeptides in are shown. Strains in which MDR012-type polypeptides are encoded by *acuI* genes that are close to *dmdA* or to the various *ddd* genes are highlighted in yellow and the two MDR028 gene products near *dddQ* in green. Those cases where the cloned *acuI* genes were shown experimentally to correct the acrylate sensitivity of the *E. coli* YhdH^−^ mutant are underlined. Other examples illustrate the wide taxonomic range of bacteria that harbour AcuI homologues, several of which are closely related to those encoded by genes linked to *ddd* or *dmdA*, and include some genera (*Xanthomonas*, *Streptomyces*, *Geobacter*) in which only some strains contain AcuI homologues. (C./V. = Chlamydiae/Verrucomicrobia; α-, β-, δ-, γ-, ε- refer to the corresponding sub-phylum of Proteobacteria). Three strains have two separate AcuI homologues, as indicated for *Dechloromonas aromatica, Alkalilimnicola ehrlichii* and *Psychrobacter cryohalolentis*. The tree with the highest log likelihoood is shown. The percentage of trees in which the associated taxa clustered together is shown next to the branches. Initial tree(s) for the heuristic search were obtained automatically using maximum parsimony method or by the BIONJ method with MCL distance matrix. The tree is drawn to scale, with branch lengths measured in the number of substitutions per site.

We found that all 37 of the searchable, genome-sequenced Roseobacter strains listed in “Roseobase” (http://www.roseobase.org/), contained a single *acuI* gene, whose products mostly ranged from 44%–57% identity to AcuI of *Rhodobacter sphaeroides* (bit score >289); that of *Citreicella* sp. SE45 was more closely related, being 85% identical (Figure 2). Of these Roseobacter strains, 26 contain the DmdA demethylase and in all but two of these cases, *acuI* was immediately 3′ of, and likely co-transcribed with, *dmdA*. In the two exceptions (*Phaeobacter gallaeciensis* BS107 and *Rhodobacterales* bacterium HTCC2255), *acuI* was elsewhere in the genome, and in the former of these, it was adjacent to a gene whose product is annotated as a betaine/carnitine/choline transporter (BCCT family), which resembled DddT, a DMSP transporter in other bacteria, such as *Halomonas* HTNK1 ([8]; see below). All 11 Roseobacter strains that lack *dmdA* also contain *acuI*, and in some cases (e.g. *Oceanicola granulosus* HTCC2516 and *Phaeobacter gallaeciensis* 2.10), it was next to a gene that resembled *dddT*, as in *Phaeobacter gallaeciensis* BS107 (see above).

The other major group of bacteria that contain DmdA demethylase are species of *Pelagibacter*, in the SAR 11 clade; unlike the Roseobacters, SAR11 bacteria lack any known DMSP lyases. All three genome-sequenced strains of *Pelagibacter* have close homologues of AcuI (which are ∼40% identical to AcuI of *Rhodobacter sphaeroides*), but none is closely linked to *dmdA*; in turn, none of their *acuI* genes is near any genes known to be involved in DMSP catabolism.

Turning to the linkages of *acuI* to the *ddd* genes that encode the various DMSP lyases, these reveal a number of different patterns, as follows.

#### DddD

The DddD enzyme is, so far, unique, since it generates DMS plus 3HP as the 3C catabolite, in contrast to the other DMSP lyases, which cleave DMSP into DMS plus acrylate. The *dddD* gene occurs, sporadically, in a range of Proteobacteria. In several of these, including *Marinomonas* MWYL1, in which it was first identified [19], it is clustered with other *ddd* genes with ancillary functions involved in DMSP catabolism (transport, regulation, subsequent catabolic steps).

To date, the only *dddD*-containing cluster that includes *acuI* is in *Halomonas* HTNK1 (Figure 1). Significantly, though, this γ-Proteobacterium grows on both DMSP and on acrylate as sole carbon sources [8], unlike, for example, *Marinomonas* MWYL1, which grows only on the former substrate. This ability is due to conversion of acrylate to 3HP, via the activities of the products of the *acuN* and *acuK* genes, which are also clustered with *dddD* and the other *ddd* genes in *Halomonas* HTNK1 ([8]; Figure 1). To date, all other *dddD*-containing bacteria lack *acuN* and *acuK*, anywhere in their genomes. In light of the postulated function of AcuI in conferring resistance to acrylate-mediated toxicity, the association of *acuI* with genes involved in the catabolism of this substrate is unlikely to be coincidental (see below).

In several other bacteria that contain *dddD*, there is no nearby *acuI*, but, in nearly all such cases, there is a version of *acuI* elsewhere in their genomes, unlinked to any other known gene involved in DMSP catabolism. These include representatives of the α- (e.g. *Hoeflea*), β- (*Burkholderia*) and γ- (e.g. *Marinomonas*) Proteobacteria.

Three Roseobacter strains, namely *Sagittula stellata* E-37, *Citreicella* sp. SE45 and *Rhodobacterales* bacterium HTCC2083, contain DddD homologues with predicted DMSP lyase activities. All three contain close homologues of AcuI and, in strain HTCC2083, the corresponding gene is downstream of *dmdA*, as in many other Roseobacters (see above). However, *Sagittula stellata* E-37 and *Citreicella* sp. SE45 are unusual among the Roseobacters in that they lack *dmdA* and their versions of *acuI* are not near any genes that are known to be involved in DMSP catabolism.

One final point concerning *dddD*-containing bacteria is that those strains in the family Rhizobiaceae that contain this gene, namely *Rhizobium leguminosarum* WSM2304, *Sinorhizobium fredii* NGR234 and *Agrobacterium tumefaciens* 5A, lack any detectable *acuI* homologue anywhere in their genomes.

#### DddY

The DMSP lyase product of *dddY* differs from the other five DMSP lyases in that it is located in the bacterial periplasm, not the cytoplasm. First described in the β-Proteobacterium *Alcaligenes faecalis* M3A [20], [21], *dddY* also occurs occasionally in other sub-phyla, namely *Arcobacter nitrofigilis* DSM7299 (ε sub-phylum), *Desulfovibrio acrylicus* W218 (δ) and in the γ-Proteobacteria *Ferrimonas balearica* DSM9799 and several different species of *Shewanella* [20].

The *Alcaligenes faecalis* M3A *acuI* gene is immediately 5′ of and co-transcribed with *dddY*, under the control of an acrylate-inducible promoter [20]. The cluster that contains these two genes resembles that of *Halomonas* HTNK1, since it includes regulatory genes (*dddZ* and *dddR*), downstream catabolic genes (*dddA, dddC*) and the *acuN* and *acuK* genes, whose products act on the acrylate, either supplied exogenously, or generated by cleavage of DMSP by the DddY lyase (Figure 1).

The genome of the ε-Proteobacterium *Arcobacter nitrofigilis* has two *dddY* genes, one of which (locus tag Arnit_0113) lies immediately 5′ of an *acuI*-like gene (Arnit_0112), whose product is in the MDR012 family, though somewhat divergently related to the AcuI sequences of other bacteria (Figure 2; see below)

Of the nine species of *Shewanella* that contain *dddY*, eight have a nearby version of *acuI* (Figure 1) the exception being *S. frigidimarina*, whose *acuI* gene is elsewhere in the genome (see below).

#### DddL

The DddL DMSP lyase cleaves DMSP into DMS plus acrylate. To date, *dddL* is largely confined to strains of bacteria in the Rhodobacterales and Rhizobiales families, with one representative (*Marinobacter* sp. MnI7-9) in the γ-Proteobacteria. All these strains contain *acuI* somewhere in their genomes, but the only known example in which it is closely linked to *dddL* is in the originally described *Rhodobacter sphaeroides* strains 2.4.1, ATCC17029 and WS8N, in which these two genes are co-transcribed ([11], [12]; Figure 1).

#### DddP

The *dddP* gene encodes a DMSP lyase in the M24 family of metallo-peptidases and is frequently found in the Roseobacters, with the majority (24 out of 37) of the genome-sequenced strains harbouring this gene [6], [22]. As described above, the *acuI* gene of most strains in this clade is 3′ of *dmdA*, and in no case was there tight linkage between *acuI* and *dddP* in any of the Roseobacters. There is evidence for horizontal gene transfer of *dddP* to some γ-Proteobacteria (*Vibrio orientalis* and *Oceanimonas doudoroffii*, the latter which has two different *dddP* genes [23]), to *Candidatus* Puniceispirillum marinum (a member of the abundant SAR116 clade of α-Proteobacteria), and even to some Ascomycete fungi [22]. In strains of *Candidatus* P. marinum, *acuI* lies upstream of *dddP* (Figure 1), the only case in which *dddP* and *acuI* are closely linked in any known bacterium.

#### DddQ

The DMSP lyase encoded by *dddQ* is confined, so far, to the Roseobacters, in which it occurs in seven different strains [6]. In none of these is *dddQ* near an *acuI*-like gene, but we did note that the *dddQ* of *R. pomeroyi* DSS-3 and the adjacent *dddQ1* and *dddQ2* genes in *Roseovarius nubinhibens* ISM [24] were closely linked to and likely co-transcribed with a gene (SPO1593 in *R. pomeroyi* DSS-3 and ISM_14095 in *Roseovarius nubinhibens*) whose product was in the MDR super-family. However, this polypeptide was predicted to be in the MDR028 sub-family and is markedly divergent to the MDR012-type AcuI polypeptide described here (Figure 2 and see below).

#### DddW

To date, *dddW* is only seen in two Roseobacter strains, namely *R. pomeroyi* DSS-3 and *Roseobacter* sp. MED193 [25]; in neither of these was it linked to an *acuI*-like gene.

### Widespread distribution of AcuI-like gene products in Bacterial phyla

Apart from those MDR028 versions that are near *dddQ*, all the *acuI*-like genes that are tightly linked to the various *ddd* and *dmdA* genes encode MDR012 sub-family medium chain reductase/dehydrogenases, as judged by the sequence similarities of their products to at least one member of this sub-group, listed by Hedlund *et al.* [14]. However, it is clear that these AcuI proteins do not comprise a particular out-group that is distinct from many other MDR012 polypeptides that occur in other bacteria, in a range of taxa, and which have no known link with DMSP and/or acrylate catabolism. This is illustrated in Figure 2, which shows a maximum likelihood tree of a range of different AcuI-like polypeptides; the gene products that were included in the tree were chosen either because they had been shown, above, to correct the acrylate sensitivity of AcuI^−^ and/or YhdH^−^ mutants, or had sequences that closely resembled such ratified polypeptides, or because their corresponding genes were closely linked to *ddd* or *dmdA* genes or, finally, because they were from a range of different Bacterial phyla. Some of these are described in more detail, below.

With the exception of *Citreicella* sp. SE45 (see above) the AcuI polypeptides of the Roseobacters are all very similar to each other (∼80% identical) whether their *acuI* gene is downstream of *dmdA* or not. Their closest matches are in bacteria with no known role in DMSP catabolism, including *Polymorphum gilvum* SL003B-26A1 (α-Proteobacterium) and *Thioalkalivibrio sulfidophilus* (γ-Proteobacterium), whose homologues are respectively ∼80% and ∼66% identical to the AcuIs in the Roseobacters. Of the AcuI polypeptides encoded by the loci near the various *ddd* genes, the Roseobacter AcuIs are more similar (∼60% identical) to the product of the *acuI* that adjoins *dddP* in *Candidatus* Puniceispirillum marinum IMCC1322 than to those that are linked to any of the various *dddY* genes and to *dddD* of *Halomonas*. And, as shown in Figure 2, the AcuI of the Roseobacters, exemplified by that of *R. pomeroyi*, is closely related to those of *Candidatus* Pelagibacter ubique, and the eponymous YhdH polypeptide of *E. coli*.

The original AcuI of *Rhodobacter sphaeroides* strain 2.4.1 more closely resembles that of *Citreicella* sp. SE45 (86%) and some other, more taxonomically distant strains, such as the β-Proteobacterium *Pusillimonas* sp. T7-7 and the Verrucomicrobiae bacterium strain DG1235 (∼65%) than it does to the polypeptides encoded by the *acuI* genes near *dmdA* of the Roseobacters (53%) or *dddY* of *Alcaligenes* (60%) or *dddD* of *Halomonas* HTNK1 (62%) (Figure 2). In turn, the respective AcuI products in these last two strains most closely resemble homologues in organisms with no links with DMSP, namely the β-Proteobacterium *Dechloromonas aromatica* RCB (78% identical) and the γ-Proteobacterium *Alkalilimnicola ehrlichii* MLHE-1 (61%), two species that are unusual in having two separate genes for AcuI-like polypeptides (Figure 2).

The sequences of the AcuI polypeptides in all but one of the *Shewanella* strains that harbour *dddY* form a closely related group, with 75–94% identify to each other, and closely resembling YhdH of *E. coli*. The exception, *Shewanella frigidimarina*, is the only one whose *acuI* gene is not linked to *dddY*; its AcuI polypeptide is rather different to the others in this genus and is closely related to that of *Halomonas* HTNK1 (Figure 2).

Finally, the product of the *acuI* gene Arnit_0112 that abuts *dddY* in *Arcobacter nitrofigilis* [26] is less closely related to the polypeptides encoded by any of the other *acuI*s that are linked to *ddd* or *dmdA* genes, and range from 37% identity (to those in the Roseobacters) to 43% (in *Halomonas* HTNK1). As with the examples above, the closest homologues to the Arnit_0112 gene product more closely resemble homologues in strains that are not involved in DMSP catabolism, such as *Psychrobacter cryohalolentis* K5 (ã-Proteobacterium; 69% identical).

These observations show clearly that although the AcuI-like polypeptides encoded by genes near *dddD, dddL, dddP*, *dddY* and *dmdA* of different bacteria are all within the MDR012 sub-family, their relatedness to each other is not necessarily congruent with either the taxonomic status of the organisms, or with the particular class of Ddd DMSP lyase encoded by the neighboring genes. For example, the AcuI's of *Rhodobacter sphaeroides* (α-Proteobacterium, DddL), *Alcaligenes faecalis* M3A (β-Proteobacterium, DddY) and *Halomonas* HTNK1 (γ-Proteobacterium, DddD) are more closely related to each other than they are to those encoded by the genes downstream of *dmdA* in the Roseobacters or next to *dddP* in *Candidatus* Puniceispirillum marinum (Figure 2).

It was also apparent from the examples above that AcuI-type polypeptides in the MDR012 sub-family occur in a very wide range of bacteria. This was further demonstrated when we used BLASTP to interrogate the current NCBI listing of genome-sequenced microbes, using AcuI of *Rhodobacter sphaeroides* as the *in silico* probe. Close homologues were present in many Bacterial phyla, with that of *Arcobacter* being the least similar (36% amino acid sequence identity; bit score 211) as illustrated in Figure 2. However, MDR102 family members were absent from all Archaeal strains currently available, and from all but two eukaryotes.

Within the Proteobacteria, MDR012 proteins are highly prevalent, though not universal among the γ-sub-phylum. Thus, although all known, genome-sequenced Enterobacteriaceae (including *Escherichia coli* – see below) and Vibrioonales contain an *acuI* homologue, it is missing from the Pasteurellales, including *Haemophilus* species. Interestingly, some individual γ-Proteobacterial genera include species that contain (e.g. *Xanthomonas gardneri*) or lack (*X. campestris* pv. *campestri*s) a polypeptide of the MDR012 sub-family. Similarly, in the α-, β-, δ- and ε-sub-phyla of Proteobacteria, *acuI* was present in only some species within a family (e.g. the *Rhizobiaceae* {α}), or even a genus (*Burkholderia* {β} or *Geobacter* {ε}). Thus, *Rhizobium leguminosarum* contains *acuI*, but strains of the closely related *Sinorhizobium*, including NGR234, which harbours *dddD* (see above) lack a close homologue. Intra-genus diversity also occurs in other bacterial phyla; the much-studied *Streptomyces coelicolor* lacks AcuI, but other strains in this Actinomycete genus contain a homologue that closely resembles those described above – the homologue in *Streptomyces* sp. SA3_actG is 70% identical to that in *Halomonas*, for example (see Figure 2). It was also apparent that some Bacteria, for example the Chlamydiae, Cyanobacteria and Spirochaetes, have few or no strains with close homologues of AcuI.

Intriguingly, there are also close AcuI homologues (∼50% identical to *Rhodobacter* AcuI) in two marine animals, namely the Tunicate sea-slug *Oikopleura dioica* and the Cnidarian *Clytia hemisphaerica*. For both species, these matches were seen to EST sequences, showing that their *acuI*-like genes are expressed. In the case of *O. dioica*, whose small (70 Mb) genome has been sequenced [27], it was possible to deduce that the corresponding gene (BACOIKO008_47) has two introns, precluding any possibility that this sequence arose via bacterial contamination and strongly pointing its acquisition by inter-Domain horizontal gene transfer.

### The AcuI homologue YhdH in *Escherichia coli* protects against the toxic effects of acrylate

The finding that mutations in *acuI* of *Rhodobacter sphaeroides* caused hypersensitivity to the inhibitory effects of acrylate [11] was the first reported phenotypic effect of mutations in any gene that encoded a medium chain reductase/dehydrogenase in the MDR012 sub-family in any bacterium. It therefore was of interest to see if mutations in similar genes in other bacteria conferred a similar phenotype. One such gene (see above) was *yhdH* of *E. coli* K-12, whose function was previously unknown.

**Figure 3:**
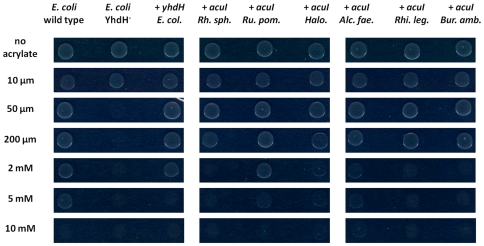
Effects of different *acuI genes* on the inhibitory effects of acrylate on the growth of *Escherichia coli*. Overnight cultures of wild type *E. coli* strain BW25113, its YhdH^−^ mutant derivative JW3222-1 and derivatives of JW3222-1 containing the cloned *yhdH* gene of *E. coli* (*E. col.*) or the *acuI* genes of *Rhodobacter sphaeroides* (*Rh. sph.*), *Ruegeria pomeroyi* DSS-3 (*Ru. pom.*), *Halomonas* HTNK1 (*Halo*), *Alcaligenes faecalis* M3A (*Alc. fae.*), *Rhizobium leguminosarum* 3841 (*Rhi. leg.*) or *Burkholderia ambifaria* AMMD (*Bur. amb.*) were spotted (10 µl) onto M9 minimal medium agar, with glycerol as the carbon source, plus acrylate at concentrations shown. Plates were incubated at 37°C for 20 hours.

We therefore obtained a YhdH^−^ insertional mutant strain JW3222-1, and compared it with its wild type *E. coli* K-12 parental strain BW25113 by growing both these strains on M9 minimal agar plates containing glycerol as carbon source, plus varying concentrations of acrylate. As shown in Figure 3 the YhdH^−^ mutant was extremely sensitive to acrylate compared to the wild type, being unable to grow at concentrations as low as 50 µM, some 100–fold lower than the concentration that was tolerated by the wild type.

**Figure 4:**
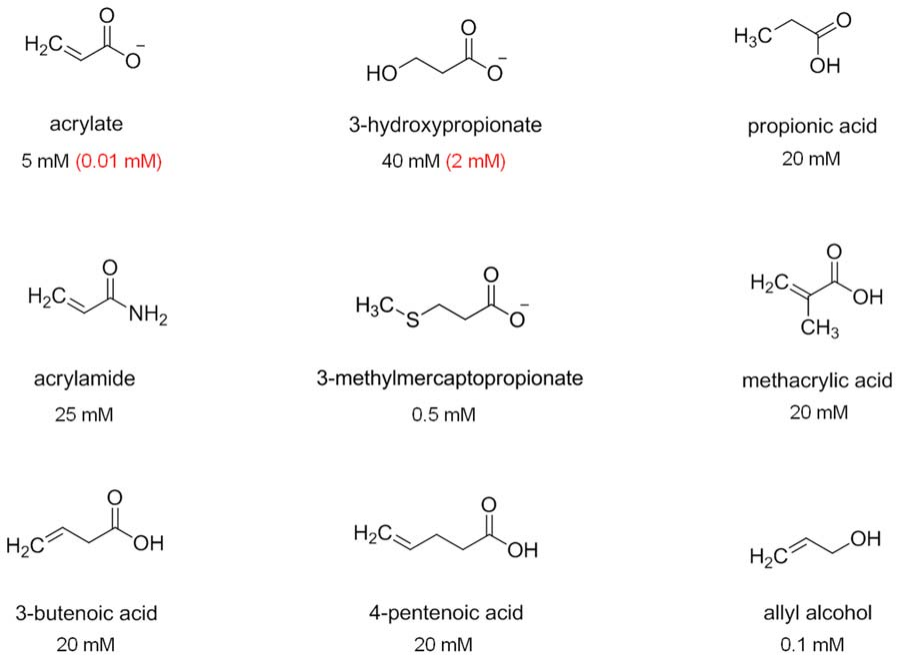
Chemical formulae of agents tested for their effects on the growth of *Escherichia coli.* Values below each chemical indicate the highest concentrations tested at which the *E. coli* wild type strain would grow. Where there was a difference between the wild type and the YhdH^−^ mutant strain, the maximum concentration at which the mutant would grow is shown in brackets.

We examined if the *yhdH* mutation affected growth in the presence of other compounds with structural similarities to acrylate and/or which might be metabolically converted to or from acrylate; namely MMPA, propionic acid, 3HP, methacrylic acid, 3-butenoic acid, 4-pentenoic acid, acrylamide and allyl alcohol (Figure 4). Of these, the only one in which the wild type and the YhdH^−^ mutant differed in their responses was 3HP. Although less inhibitory to both strains than acrylate, the wild type tolerated 40 mM 3HP in the medium, but the YhdH^−^ mutant failed to form colonies at 5 mM.

We also exposed the wild type and YhdH^−^ mutant *E. coli* strains for 24 hours to high levels (10 mM) acrylate in M9 buffer that lacked any other carbon source and which therefore did not support cell growth. Following this treatment, the cells were washed, serially diluted, and plated on LB complete media and incubated, before counting the numbers of colonies. Compared to the control (buffer with no acrylate), exposure to acrylate did not affect the survival of either strain. Thus, the effects of acrylate appear to be bacteriostatic rather than bacteriocidal, and/or this observation may mean that acrylate must be transformed to the genuine inhibitory compound and that this only occurs in actively metabolising cells.

### Correction of the acrylate sensitivity of an *E. coli* YhdH^−^ mutant with cloned *acuI* from other bacteria

The finding of such a clear phenotypic difference in acrylate tolerance in the wild type and a YhdH^−^ mutant of *E. coli* offered a facile way to establish if this phenotype could be corrected by the cloned *acuI*-like genes from other organisms. We therefore amplified and cloned the individual *acuI* genes from genomic DNA of *E. coli* itself and from several bacteria whose *acuI* gene was closely linked to different *ddd* genes or to *dmdA*. These were *Halomonas* HTNK1 (next to *dddD*), *Alcaligenes faecalis* M3A (*dddY*), *Rhodobacter sphaeroides* (*dddL*), *Arcobacter nitrofigilis* (*dddY*), *Burkholderia ambifaria* (which has a distantly linked *dddD* gene [19]) and *R. pomeroyi* DSS-3 (*dmdA*). We also examined the effects of the cloned *acuI* gene of *Rhizobium leguminosarum* 3841, a strain with no known links to DMSP catabolism, whose pRL120182 gene encodes a polypeptide that is 53% identical to AcuI of *Rhodobacter sphaeroides*. The resultant recombinant plasmids were each transformed into the YhdH^−^ *E. coli* mutant JW3222-1, and the transformants were tested for acrylate tolerance, in comparison with the wild type *E. coli* strain BW25113. As shown in Figure 3, all the clones tested could restore acrylate resistance to the *E. coli* YhdH^−^ mutant.

In contrast, a plasmid that contained the *Roseovarius nubinhibens* ISM gene (ISM_14095) in the *dddQ* cluster of that strain and which encodes an MDR028-type polypeptide (see above), failed to correct the acrylate sensitivity of the *E. coli* YhdH^−^ mutant.

### Repercussions of the close linkage of *acuI* and *dmdA* in the model Roseobacter *Ruegeria pomeroyi* DSS-3

*R. pomeroyi* DSS-3 was the first Roseobacter to be genome-sequenced [28] and has had a relatively long history as a subject for studies on DMSP catabolism. Earlier biochemical studies showed that it can both demethylate DMSP and cleave it to acrylate plus DMS, with the former pathway being relatively more important at lower DMSP concentrations [17]. Genetic analyses then showed that the demethylation pathway was mediated by the DmdA demethylase, with the subsequent, sequential downstream catabolic reactions being catalysed by the products of the *dmdB, dmdC* and *dmdD* genes [9], [10]. This strain also has no less than three DMSP lyases, DddP, DddQ and DddW, all of which contribute to the cleavage of DMSP to acrylate plus DMS [23].

As stressed by Reisch *et al.* [7], any “switch” that modulates the partitioning of the fluxes through the cleavage and demethylation pathways may be important, not only for the individual strains and species of Roseobacters, but more widely, with significant environmental consequences. This is because the volatile DMS product of the DMSP lyases is a major vehicle to transfer sulfur from the seas to the atmosphere, thence back to land [29]. The finding in many Roseobacter strains that *dmdA*, a key gene in the demethylation pathway is immediately 5′ of *acuI*, which is implicated in handling acrylate, a major product of the cleavage pathway, suggests that there may be some intimate links between these two catabolic routes in the Roseobacters, which may impinge on this switch.

To further examine these links, we first generated insertional mutations into *dmdA* and into *acuI* of *R. pomeroyi* DSS-3 (see Materials and Methods). In light of the hypersensitivity of the AcuI^−^/YhdH^−^ mutants of *Rhodobacter sphaeroides* and *E. coli*, it was not unexpected to find that the insertion into *acuI* of *R. pomeroyi* conferred a similar phenotype. This mutant failed to grow on minimal medium containing 0.2 mM acrylate, with or without the alternative carbon source succinate, whereas wild type *R. pomeroyi* was unaffected for growth at acrylate concentrations greater than 5 mM.

Since catabolism of DMSP by one or more of the *R. pomeroyi* Ddd DMSP lyases generates acrylate as one of the products, we also examined if the mutation in *acuI* affected the growth of *R. pomeroyi* when DMSP was in the medium. The wild type tolerated concentrations greater than 20 mM, but growth of the AcuI^−^ mutant was strongly inhibited by this concentration of DMSP.

The insertional mutation into *dmdA* of *R. pomeroyi* led to similar phenotypes as those in the AcuI^−^ mutant. Thus, growth of the DmdA^−^ mutant was strongly inhibited on medium that contained acrylate as low as 0.5 mM and on DMSP at 1 mM even in the presence of the “regular” carbon source, succinate. There are two likely contributing factors to explain why the *dmdA* mutation would cause hypersensitivity to acrylate and to DMSP. First, the insertion into *dmdA* is predicted to be polar on the expression of the downstream *acuI*, which would abolish or reduce the AcuI-mediated protective effects against acrylate, either added exogenously, or generated by the cleavage of DMSP. Secondly, the DmdA^−^ mutant would be expected to channel more of the DMSP catabolic flux through one or more of the DMSP lyases, since the demethylation pathway was blocked. Indeed, it was directly shown by Howard *et al.* [9], that a DmdA^−^ mutant of this strain produced more acrylate from DMSP than the wild type. Consistent with this, we noted that production of DMS, the other product of the cleavage pathway, was also enhanced, ∼5-fold, when our DmdA^−^ mutant was assayed for DMSP lyase activity.

Further evidence for these explanations was obtained from a series of complementation tests, using a series of separate plasmid constructions as follows. We cloned the *R. pomeroyi acuI* and *dmdA* genes, both individually and together, into the wide host-range cloning vector pBIO1878 [25], and separately introduced the resultant recombinant plasmids into the *R. pomeroyi* AcuI^−^ and DmdA^−^ mutants in tri-parental conjugational matings. The acrylate and DMSP sensitivities of both mutants were corrected by the plasmids that contained *acuI* alone (pBIO2024) or in tandem with *dmdA* (pBIO2022) but, significantly, the plasmid that contained *only dmdA* (pBIO2021) did not overcome the sensitivity to either of these compounds. Thus, the sensitivities to both acrylate and to DMSP in the DmdA^−^ mutant must be due to the effect of the insertional mutation on the expression of the downstream *acuI* gene.

We noted a second link between acrylate and the *R. pomeroyi dmdA-acuI* operon, which concerns its transcriptional regulation. In the course of a microarray experiment, the expression levels of both *acuI* and *dmdA* were substantially enhanced (∼14-fold) when the cells were grown in MBM minimal media in the presence of 5 mM DMSP. Significantly, when 5 mM acrylate was present in the growth medium, the expression of these two genes was also induced, and by a similar factor (∼12-fold). This marked induction of *dmdA* and of *acuI* by DMSP was not seen in a different transcriptomic analysis of this strain by Bürgmann *et al.* [30], most likely because of the much lower concentration (80 µM) of DMSP that was added to the medium in that study.

To examine the expression of the *R. pomeroyi* DSS-3 *dmdA-acuI* operon in more detail, we made two *lacZ* transcriptional fusion plasmids, both based on the wide host-range reporter vector pBIO1878 (See Figure 1). Both plasmids contained the promoter region of the *dmdA-acuI* operon, but their 3′ ends, fused to the *lacZ* reporter, were either in *dmdA* (pBIO2020) or *acuI* (pBIO2021). These two plasmids were each mobilised into *R. pomeroyi* DSS-3 by conjugation, and cultures of the transconjugants were each grown in minimal medium, containing or lacking either acrylate or DMSP (each at 5 mM) before assaying their β -galactosidase activities. The results tallied with those in the microarrays; the expression of both genes was markedly enhanced by both compounds compared to the levels in the control medium, with a ∼10-fold increase with DMSP and ∼14-fold when acrylate was in the growth medium. Thus, for the *acuI-lacZ* fusion, the β -galactosidase activities were 56±3 Miller Units in the control medium, 502±20 in the +DMSP and 780±32 in the +acrylate media. The finding that a breakdown product of the DMSP cleavage pathway can induce the production of the DMSP demethylase DmdA means that there is a regulatory tie-in between the two pathways, with possible physiological consequences (see below).

## Discussion

Interest in the *acuI* gene stemmed from its close linkage to a range of different *ddd* and *dmdA* genes that are involved in the initial steps of DMSP catabolism in a wide range of bacteria. A more direct link with DMSP, via one of its catabolites, acrylate, came from observations on the *acuR-acuI-dddL* operon of *Rhodobacter sphaeroides*; not only was its expression enhanced by the co-inducer acrylate, but AcuI^−^ mutants were less effective in the breakdown of acrylate and were more sensitive to the growth-inhibiting effects of this compound [11].

However, the bioinformatically and experimentally based observations described here show that the role of the *acuI* gene extends far beyond the realm of DMSP catabolism and DMSP-catabolising bacteria. Furthermore, the recent demonstration that AcuI of *Rhodobacter sphaeroides* has acryloyl-CoA reductase activity, which converts acryloyl-CoA to propionyl-CoA [13], provides significant, and highly relevant biochemical insights. Taken together, these new data prompt us to propose a novel, and potentially widespread, functional role for the AcuI-type gene products, as follows.

In general terms, we suggest that the intracellular presence of a compound that can be formed endogenously, but whose production is elevated in cells that are grown with exogenous acrylate, is extremely inhibitory to growth. The primary role of AcuI is to act as a “cleansing agent” to reduce the concentrations of this compound to sub-inhibitory levels. Given the enzymatic activity of AcuI, the most likely suspect for this molecule is acryloyl-CoA itself, whose cellular toxicity was alluded to by Herrmann *et al.* [31]. Here, we consider the evidence in favour of this model.

**Figure 5:**
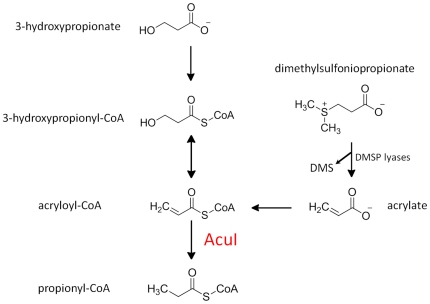
AcuI-mediated conversion of acryloyl-CoA to propionyl-CoA. The pathway from 3-hydroxypropionate to propionyl-CoA is adapted from Schneider *et al.* [13]. Also shown is how DMSP lyases can generate acrylate, which is postulated to be converted to acryolyl-CoA by a CoA-ligase, as yet unidentified. The exact identity of the DMSP lyase is strain-dependent; e.g., *Rhodobacter sphaeroides* has the DddL lyase, and in *Ruegeria pomeroyi*, the acrylate can be generated from DMSP by DddP, DddQ and/or DddW.

First, and most obviously, it explains why mutations in *acuI* of *Ruegeria, Rhodobacter* and in the equivalent gene, *yhdH*, in *E. coli* are hypersensitive to the inhibitory effects of acrylate. Of the other compounds tested, the AcuI^−^/YhdH^−^ mutants were also sensitive to 3-OH-propionate, consistent with its conversion to 3-OH-propionyl-CoA, thence to acryloyl-CoA, as suggested by Schneider *et al.* ([13]; see Figure 5). The fact that these hypersensitive phenotypes of the *E. coli* YhdH^−^ mutant were corrected by the cloned *acuI* genes from a range of different bacteria confirmed that these genes, too, are all functionally equivalent.

Acryloyl-CoA is predicted to be a very strong electrophile that would react with other important molecules in the cell [31]. Although it was not demonstrated formally that acryloyl-CoA is indeed the culprit that is responsible for the growth inhibition in acrylate-grown cells (and, less so, in response to 3HP), there is a precedent in which a related molecule, propionyl-CoA, was reported to be a “suicide substrate” that inactivated a short-chain acyl-CoA dehydrogenase, due to modification of its flavin adenine dinucleotide (FAD) cofactor [32]. It is unlikely that the inhibitory effects seen with acrylate are due solely to the interaction of acryloyl-CoA with the FAD of bacterial acyl-CoA dehydrogenase itself, since mutations in the *E. coli fadE* gene that encodes this enzyme are capable of normal growth, unless forced to use a fatty acid such as oleate as a carbon course [33]. However, if the more reactive acryloyl-CoA did react with FAD of other enzymes that contain this cofactor, or its reduced FADH_2_ form, then this might form the basis of its inhibitory effects if such enzymes include any that are needed for normal growth.

One implication of this model is that many bacteria (including *E. coli* K-12) can convert exogenous acrylate to acryloyl-CoA. There have been no reports of such an activity in *E. coli*, and, to our knowledge this bacterium does not encounter acrylate in its natural environment. The same holds true for many of the other bacteria, in a range of different taxa, that also harbour close homologues of AcuI. It therefore seems unlikely that *E. coli* has a dedicated acryloyl-CoA ligase activity. However, at least one acetyl-CoA ligase enzyme has broad specificity and can attach CoA to other substrates, including acrylate [34]. Indeed, we have noted that when 1 mM ^14^C acrylate was fed to mid-log phase cultures of *E. coli* BL21, ca. 75% of the counts appeared as ^14^CO_2_ after 16 hours incubation, with concomitant loss of the original labelled substrate (M.J. Sullivan, unpublished). So, *E. coli*, and perhaps an unexpectedly wide range of bacteria, may be able to catabolise acrylate *in vivo*, albeit rather inefficiently.

Concerning the linkage of *acuI* with several different classes of DMSP catabolic genes in different bacteria, we propose that this is an adaptive response in which the AcuI enzyme may counter the inhibitory effects of the acrylate that is obtained directly from the environment and/or which is made by cleavage of DMSP by Ddd lyase(s). Although the terms “acrylate” and “acrylic” are in common parlance, this is largely because these are widely used feed-stocks in the petrochemical industry. To our knowledge, the only natural environments in which there are significant amounts of acrylate are those, such as coral reefs, with very high levels of DMSP, much of which is converted to acrylate by microbial action [35], [36].

As shown above, though, the *acuI* gene of many DMSP-catabolising bacteria is not linked to the DMSP catabolic genes. However, the features and distributions of those *ddd/dmdA* genes that do or do not have a closely linked *acuI* gene are informative. Most notably, the only case in which *acuI* is near the *dddD* gene is in *Halomonas* HTNK1, and this is the only known *dddD* cluster that also includes the *acuN* and *acuK* genes. Significantly, DddD is the only known DMSP lyase that does not generate acrylate as its C3 product, so, in most bacteria that harbor *dddD*, there is no need for any dedicated protection system from the damaging effects of acrylate and its subsequent catabolite(s). However, the possession of the *acuN* and *acuK* genes confers on *Halomonas* HTNK1 the ability to catabolise exogenous acrylate, converting it to 3HP, the same initial catabolite as that generated by the action of DddD on DMSP. Furthermore, acryloyl-CoA is a predicted transient intermediate in the conversion of acrylate to 3HP, since the AcuN polypeptide is in a family of acyl-CoA transferases. Therefore, this unusual, dual-purpose *ddd/acu* gene cluster of *Halomonas* may have recruited a closely linked *acuI* gene for “added protection” [8].

Turning to DddY, another enzyme that *does* release acrylate from DMSP, there are nearby *acuI* genes in nearly all the diverse bacteria that contain this lyase, the only exceptions, to date, being in *Shewanella frigidimarina* NCIMB 400 and *Ferrimonas balearica* DSM9799, both of which have *acuI* homologues, but these are elsewhere in their genomes. In *Alcaligenes faecalis* M3A, the intimate relationship of acrylate and its *acuI* gene is further emphasised by the fact that expression of its adjacent *acuI-dddY* genes is massively increased when the cells were grown in the presence of acrylate [20].

In contrast, concerning the genes for the four other DMSP lyases, there are no *acuI*-like genes near *dddQ* and *dddW* in any bacteria to date, and only one case each in which *dddP* (*Candidatus* Puniceispirillum marinum) or *dddL* (*Rhodobacter sphaeroides*) is close to *acuI*, even though all these genes encode DMSP lyases that cleave DMSP into acrylate plus DMS. Our explanation for this is that with the exceptions of the very few cases of horizontal gene transfer, these four *ddd* genes are confined to the Roseobacters and, in the majority of genome-sequenced strains of this clade, their *acuI* gene is immediately downstream of their *dmdA* genes. The ability to catabolise DMSP, both by demethylation and by cleavage, is part of the core lifestyle of the Roseobacters [18], [37], as witnessed by the fact that the great majority of them contain both *dmdA* plus at least one *ddd* gene. However, the particular portfolio of *ddd* gene varies considerably from strain to strain [18], [23]. From an adaptive point of view, it may therefore be most efficient if *acuI* is linked to *dmdA*, so that its product can deal with the potentially harmful consequences of the acrylate that is formed by *any* of the DMSP lyases in the same strain. In the SAR11 clade, the other major group of bacteria with the DmdA DMSP demethylase, their *acuI* genes are not linked to *dmdA*, consistent with the fact that these bacteria do not cleave DMSP into acrylate plus DMS, so have no need for a specialised acrylate protection system that connects to DMSP catabolism.

The linkage and co-expression of *dmdA* and the downstream *acuI* gene may also affect the ways in which the Roseobacters partition the DMSP catabolic flux between the cleavage and the demethylation routes. The relative importance of these pathways in *R. pomeroyi* DSS-3 had been shown to be influenced by the concentrations of the DMSP substrate, with a higher relative flux through the cleavage route as the DMSP concentrations increased [17]. Furthermore, DmdA^−^ mutants produce proportionally more acrylate and DMS from DMSP than does the wild type ([9]; see above), due to the blockage of the demethylation pathways, which results in more of the substrate being available for cleavage by the DddP, DddQ and/or DddW DMSP lyases of this strain. However, our new finding that the acrylate that is produced by these lyases is itself a likely co-inducer of *dmdA* means that there is also a more direct regulatory link between the expression of the cleavage and demethylation pathways. This could have a homeostatic outcome, whereby, as the intracellular levels of acrylate rise due to lyase activities, there is increased expression of the DmdA demethylase. Thus, by increasing the flux through the demethylation pathway, less DMSP substrate is available for the acrylate-generating action of the Ddd lyases and by enhancing the levels of AcuI, there is enhanced protection against the potential damage, most likely inflicted by acryloyl CoA. Then as the acrylate levels drop, the relative inputs of the various lyases rise, with a concomitant increase in intracellular acrylate. The situation may be more complex, though, for at least two reasons.

Firstly, the expression of one of the *ddd* genes, *dddW*, is considerably enhanced in cells of *R. pomeroyi* DSS-3 grown in the presence of DMSP, but acrylate is not a co-inducer for this gene [25]. Secondly, although the demethylation pathway in *R. pomeroyi* DSS-3 that was described recently [10] does not generate any acrylate, a previously suggested catabolic scheme proposed that MMPA, the initially demethylated product of DMSP, is subject to demethiolation in a step that would yield acrylate plus methane thiol [38], [39]. Since this has not been formally precluded as method for DMSP catabolism, albeit a minor one, it is possible that some of the acrylate that acts as a co-inducer of the *dmdA-acuI* operon may have originated by this route.

The very fact that a catabolic *product* (in this case, acrylate) enhances the expression of an enzyme that acts on the *substrate* (DMSP) is, in itself, an unusual phenomenon in bacterial gene regulation. However, this mode of control is a feature of bacterial catabolism of DMSP and was first noted in physiological experiments, in which pre-growth of different bacteria in the presence of DMSP or 3HP enhanced their levels of DMSP lyase activity [5]. Since then, these DMSP catabolites were shown to be co-inducers of *ddd* catabolic genes in other bacteria, such as *Halomonas* and *Alcaligenes* [6]. Indeed, in *Rhodobacter sphaeroides*, although the substrate DMSP appeared to induce its *dddL* gene, the DMSP had to be cleaved by the DddL lyase, forming the *bona fide* co-inducer, acrylate [11]. It is striking that this phenomenon of catabolite-responsive gene induction extends to the *dmdA* gene involved in the demethylation pathway. Prompted by this observation, we also investigated if MMPA, the demethylated derivative of DMSP generated by the action of the DmdA demethylase was a co-inducer of the *dmdA-acuI* operon; however, there was no induction of the *dmdA-lacZ* or *acuI-lacZ* fusions when strains of *Ruegeria* carrying the corresponding reporter plasmids were pre-grown in the presence of 5 mM MMPA.

It is noteworthy that many bacteria do not have close homologues of AcuI in their deduced proteomes. These may be entire clades, such as the Chlamydiae Phylum or the Order Pasteurellales within the γ-Proteobacteria. Perhaps more strikingly, there are also several cases in which individual genera include some strains that do and some that do not contain *acuI*. Indeed, we demonstrated directly that the cloned pRL120182 gene of *Rhizobium leguminosarum* 3841, whose product is a predicted AcuI enzyme, conferred acrylate resistance to the *E. coli* YhdH^−^ mutant. Yet, a very closely related strain, *Sinorhizobium fredii* NGR234, lacks an AcuI homologue but in our hands (unpublished) was as tolerant of acrylate in the medium as *R. leguminosarum* 3841.

Do these various strains that lack AcuI have alternative methods to circumvent the toxic effects of acrylate? In that connection, Hetzel *et al.* [40] purified a heterotrimeric enzyme with acryloyl-CoA reductase activity from *Clostridium propionicum*, but the N-terminal sequences of none of the polypeptides resembled that of AcuI. Similarly, the conversion of acryloyl-CoA to propionyl-CoA by two other MDR family members has been reported in the Crenarchaeota, *Sulfolobus tokodaii* [41] and the green non-sulfur bacterium, *Chloroflexus aurantiacus* [42]. Both strains engage in CO_2_ fixation, using a cycle that includes the conversion of acryloyl CoA to propionyl-CoA. In *Chloroflexus*, this is mediated by two adjacent MDR-type domains within a multi-functional protein, but in the Archaea, this reductive step was performed by a stand-alone MDR polypeptide. However, the sequence similarities of both these enzymes to the MDR012 class of AcuI/YhdH polypeptides studied here is very limited.

Although *acuI* was first of interest because of its link with acrylate in bacteria that synthesised this molecule via DMSP catabolism, this is just one aspect of a wider and more important role for this gene and its close relatives. This even extends to the allocation of a function to an *E. coli* gene whose current description is “Putative quinone oxidoreductase, function unknown” (*“ecogene”*; http://ecogene.org/geneinfo.php?eg_id=EG11315). Although the work described here includes the first example of a phenotype that can been ascribed to mutations in the *E. coli yhdH* gene, the molecular basis of the inhibitory effects of acrylate remain to be formally confirmed as, indeed, does the proposal that acryloyl-CoA is the toxic molecule. It will also be of interest to establish if other enzymes, which do not resemble AcuI in their sequence, but which mimic its role in conferring acrylate resistance, occur in other organisms that do not have a recognisable *acuI* gene in their genomes.

## Materials and Methods

### Bacterial strains, plasmids and media

Strains and plasmids used in this study are listed in Table S1. *E. coli* was grown at 37°C on Luria-Bertani (LB) or M9 minimal media with 10 mM glycerol as the regular carbon source [43]. *Ruegeria pomeroyi* DSS-3 was grown at 28°C on ½ YTSS [44] or MBM minimal medium with 10 mM succinate as carbon source (see [45]). Antibiotics were used at the following concentrations (µg ml^−1^): Kanamycin (20), Tetracycline (5), Ampicillin (100), Spectinomycin (200) and Rifampicin (20).

To assay β-galactosidase, *R. pomeroyi* cells were grown overnight in either MBM minimal medium with 10 mM succinate as carbon source and, where appropriate, the co-inducers DMSP and acrylate, each at 5 mM, prior to being assayed for β-galactosidase as described in Rossen *et al.* [46]. The transcriptional fusion plasmid vector was pBIO1878, which is based on pMP220 [47] and includes a selectable Spc^R^ cassette to facilitate selection in Roseobacters [25].

### *In vitro* and *in vivo* genetic manipulations

General handling and manipulation of DNA were done as in Wexler *et al.* [48]. Plasmids were conjugated into the Rif^R^ *R. pomeroyi* strain J470 by triparental mating using helper plasmid pRK2013 [49].

### Gene amplification and construction of plasmids and mutants

Fragments of genomic DNA containing the intact *yhdH* of *E. coli* and the *acuI* genes of a selection of other bacteria, were each amplified by PCR from genomic DNA obtained from the *E. coli* K12 strain BW25113, *Ruegeria pomeroyi* DSS-3, *Rhodobacter sphaeroides* 2.4.1 [50], *Alcaligenes faecalis* M3A [20], *Halomonas* sp. HTNK1 [8], *Rhizobium leguminosarum* 3841 [51] and *Burkholderia ambifaria* AMMD [19] using primers that contained appropriate restriction sites (Table S2). Digested PCR products were then ligated into pET16b, pET21a, pRK415 [52] or pBIO1878 and transformed into *E. coli* strain 803 [53]. For cloning *Roseovarius nubinhibens* ISM_14095, a 4 kb *Pst*I fragment from pBIO1880 was sub-cloned into pBluescript [54]. The *Arcobacter nitrofigilis acuI* gene was identified by screening a genomic library in the cosmid vector pLAFR3 [55] of this strain for any cosmids that corrected the acrylate sensitivity of the YhdH^−^ mutant.

To facilitate the cloning of the *R. pomeroyi dmdA* and *acuI* genes, together with their promoter, a 2.4 kb PCR fragment containing both these genes was first cloned into pBluescript using *Xba*I and *BamH*I sites in the primer sequences, yielding plasmid pBIO2019. To construct the *dmdA*-*lac* fusion plasmid, pBIO2019 was digested with *Xba*I and *Pst*I, releasing a 350 bp fragment that contained the *dmdA*/*acuI* promoter and whose 3′ end was within *dmdA*. This fragment was then cloned into pBIO1878 (Spc^R^/Tet^R^), forming pBIO2020. To construct an *acuI*-*lac* fusion plasmid, pBIO2019 was digested with *Xba*I and *NsiI*, releasing a 1.4 kb fragment that contained the *dmdA*/*acuI* promoter and whose 3′ end was in *acuI*. This fragment was cloned into pBIO1878 that had been digested with *Xba*I plus *Pst*I to form pBIO2021.

For the complementation tests with the AcuI^−^ and DmdA^−^ mutants of *R. pomeroyi* DSS-3, the following plasmids were constructed. The 2.4 kb fragment that contains intact *dmdA* and *acuI* plus their promoter, which was used to construct pBIO2019 (see above), was released from that plasmid and cloned into pBIO1878 to form pBIO2022. To clone *acuI* alone, though still under the control of its own promoter, plasmid pBIO2019 was first digested with *Sph*I, then religated in a procedure that removed three *Sph*I fragments internal to *dmdA*, but which leaves *acuI* intact. The deletant plasmid pBIO2023 was then digested with *Xba*I plus *BamH*I and the released fragment was sub-cloned into pBIO1878, forming pBIO2024. The plasmid that contained only *dmdA* was pBIO2021, described above. The dimensions and names of the relevant plasmids are shown in Figure 1 and Table S1. All recombinant plasmids were ratified by sequencing of the inserts, performed by Genome Enterprise Ltd, Norwich Research Park, Norwich, UK.

The insertional mutations into *acuI* and into *dmdA* of *R. pomeroyi* were made by a procedure in which fragments internal to each of the two genes were cloned, separately, into the suicide plasmid vector pBIO1879 [24], a Spc^R^ derivative of the suicide vector pK19*mob* [56]. The internal *dmdA* fragment was made by amplifying a 800 bp fragment from *R. pomeroyi* DSS-3 genomic DNA using forward and reverse primers (Table S2) which respectively contain *EcoR*1 and *Pst*I restriction sites, prior to cloning into pBIO1879, cut with the same enzymes to form pBIO1870. A 960 bp *acuI* internal DNA fragment was made by digesting pBIO2019 with *Sal*I, and this was then cloned into *Sal*I-digested pBIO1879 to form pBIO2025. The two plasmids pBIO1870 and pBIO2025 were then each conjugated to *R. pomeroyi* DSS-3 in triparental matings, selecting Rif^R^/Spc^R^/Kan^R^ transconjugants, which should arise via a single cross-over event within the corresponding genes. These mutants were confirmed by colony PCR and Southern Blotting and were termed J527 (AcuI^−^) and J471 (DmdA^−^).

The *E. coli* strains BW25113 and its *yhdH* ^−^ mutant derivative JW3222-1 were obtained from the Keio collection of *E. coli* K-12 in-frame, single-gene knockout mutants [57] through the *E. coli* Genetic Stock Center, Yale University, New Haven, Connecticut.

### Growth inhibition experiments

Starter cultures of the various *E. coli* or *R. pomeroyi* strains were grown to mid log-phase in complete medium. Cells were adjusted to equivalent OD_600_ values, washed in minimal medium. For *E. coli*, 10 µl aliquots were spotted onto plates of M9 minimal agar medium containing varying levels of acrylate, DMSP or other tester compounds. Growth was scored after incubation at 37°C for 20 hours. For *R. pomeroyi*, cells were used to inoculate 5 ml MBM minimal media containing 10 mM succinate as carbon source and varying levels (50 µM to 10 mM) of acrylate or DMSP (200 µM - 20 mM). Cultures were incubated at 28°C, with shaking and the growth levels recorded after 48 hours.

### *In silico* analysis

Sequence analysis was performed using BLAST at NCBI (http://blast.ncbi.nlm.nih.gov/Blast.cgi) and “Roseobase” (http://www.roseobase.org/). Phylogenetic trees were produced using the Maximum Likelihood method based on the JTT matrix-based model [58]. Evolutionary analyses were conducted in MEGA5 [59].

## Supporting Information

Table S1 Amp^R^, ampicillin resistant; Kan^R^, kanamycin resistant; Rif^R^, rifampicin resistant; Spc^R^, spectinomycin resistant; Tet^R^, tetracycline resistant.
(DOCX)

Table S2 Introduced restriction sites are shown by underlining.
(DOCX)
