# Supplementary material for: The Ruegeria pomeroyi acuI Gene Has a Role in DMSP Catabolism and Resembles yhdH of E. coli and Other Bacteria in Conferring Resistance to Acrylate
Source: PLoS One. 2012 Apr 26;7(4):e35947. doi: 10.1371/journal.pone.0035947 (PMC3338564; doi:10.1371/journal.pone.0035947)
Supplement: Table S1 — AmpR, ampicillin resistant; KanR, kanamycin resistant; RifR, rifampicin resistant; SpcR, spectinomycin resistant; TetR, tetracycline resistant. (DOCX) [file pone.0035947.s001.docx]

**Table S1 Strains and plasmids used in this work**

| **Bacteria** | **Characteristics** | **Source** |
| --- | --- | --- |
| *Escherichia coli* BW25113 | Wild type parent strain for the Keio collection of single gene knockouts | [57] |
| *E.* *coli* JW3222-1 | YhdH^-^ mutant of strain BW25113 from Keio collection | [57] |
| *E. coli* 803 | Met^-^; used as host for transformation with large plasmids | [55] |
| *E. coli* JM101 | Used as host for blue/white screening of cloned inserts | *New England Biolabs* |
| *Rhodobacter sphaeroides* 2.4.1 (ATCC17023) | Wild type Ddd^+^ strain | [50] |
| *Ruegeria pomeroyi* J470 | *Ruegeria pomeroyi* DSS-3 Wild type (Rif^R^) | [24] |
| *Ruegeria pomeroyi* J471 | *R. pomeroyi* J470 with an insertion in *dmdA* | This work |
| *Ruegeria pomeroyi* J527 | *R. pomeroyi* J470 with an insertion in *acuI* | This work |
| *Alcaligenes faecalis* M3A | Wild type | [21] |
| *Rhizobium leguminosarum* 3841 | Wild type | [51] |
| *Halomonas* sp. HTNK1 | Wild type | [8] |
| *Burkholderia ambifaria* AMMD | Wild type | <http://genome.jgi-psf.org/buram/buram.home.html>. [19] |
| *Arcobacter nitrofigilis* DSM 7299 | Wild type | [26] |
| **Plasmids** | **Characteristics** | **Source** |
| pBluescript SK- | Used as high copy number cloning vector (Amp^R^) | [54] |
| pRK2013 | Used as mobilising plasmid in tri-parental crosses (Kan^R^) | [49] |
| pLAFR3 | Wide host-range cosmid vector (Tet^R^) | [55] |
| pMP220 | Wide host-range promoterless-*lacZ* probe vector (Tet^R^) | [47] |
| pET16b | Expression vector (Amp^R^) | *Novagen* |
| pET21a(+) | Expression vector (Amp^R^) | *Novagen* |
| pRK415 | Wide host-range cloning vector (Tet^R^) | [52] |
| pK19mob | Suicide insertion plasmid (Kan^R^) | [56] |
| pBIO1870 | Internal fragment of *R. pomeroyi* DSS-3 *dmdA* cloned into pBIO1879 | This work |
| pBIO1878 | Wide host-range *lac* reporter plasmid, based on pMP220 (Spc^R^, Tet^R^) | [25] |
| pBIO1879 | Spc^R^ cassette cloned into pK19*mob* | [24] |
| pBIO1880 | Cosmid from *R. nubinhibens* ISM gene library containing *dddQ1*, *dddQ2* and ISM_14095 genes, cloned in pLAFR3 | [24] |
| pBIO2011 | *E. coli* BW25113 *yhdH* cloned in pET21a | This work |
| pBIO2012 | *Rhodobacter sphaeroides* 2.4.1 *acuI* cloned in pET16b | This work |
| pBIO2013 | *Ruegeria pomeroyi* DSS-3 *acuI* cloned in pET21a | This work |
| pBIO2014 | *Halomonas* sp. HTNK1 *acuI* cloned in pRK415 | This work |
| pBIO2015 | *A.* *faecalis* M3A *acuI* cloned in pMP220 | This work |
| pBIO2016 | *Rhizobium leguminosarum* 3841 *acuI* cloned in pET21a | This work |
| pBIO2017 | *Burkholderia ambifaria* AMMD *acuI* cloned in pET21a | This work |
| pBIO2019 | *R. pomeroyi* DSS-3 *dmdA* and *acuI* with their native promoter cloned in pBluescript | This work |
| pBIO2020 | *R. pomeroyi* DSS-3 *dmdA*-*lacZ* reporter fusion plasmid | This work |
| pBIO2021 | *R. pomeroyi* DSS-3 *acuI-lacZ* reporter fusion plasmid | This work |
| pBIO2022 | *R. pomeroyi* DSS-3 *dmdA* and *acuI* with their native promoter cloned in pBIO1878 | This work |
| pBIO2023 | pBIO2019 with a deletion in *dmdA* | This work |
| pBIO2024 | *R. pomeroyi* DSS-3 *acuI* with its own promoter (from pBIO2023) cloned into pBIO1878 | This work |
| pBIO2025 | Internal fragment of *R. pomeroyi* DSS-3 *acuI* cloned into pBIO1879 | This work |
| pBIO2026 | *Arcobacter nitrofigilis acuI* cloned in the cosmid pLAFR3 | This work |
| pBIO2027 | *Roseovarius nubinhibens* ISM ISM_14095 cloned into pBluescript | This work |
