# Supplementary material for: The Ruegeria pomeroyi acuI Gene Has a Role in DMSP Catabolism and Resembles yhdH of E. coli and Other Bacteria in Conferring Resistance to Acrylate
Source: PLoS One. 2012 Apr 26;7(4):e35947. doi: 10.1371/journal.pone.0035947 (PMC3338564; doi:10.1371/journal.pone.0035947)
Supplement: Table S2 — Introduced restriction sites are shown by underlining. (DOCX) [file pone.0035947.s002.docx]

**Table S2 Oligonucleotide primers used in this study**

| **Primer** | **Sequence** | **Used for** |
| --- | --- | --- |
| yhdHNdeFOR1 | 5'-GCACATGAGGACCACATATGCAGGCG-3' | For cloning fragment used in pBIO2011 |
| yhdHBamREV1 | 5'-CCAGTTTGGATCCTGTTGTATAACTTCC-3' | For cloning fragment used in pBIO2011 |
| RsphacuINdeFOR1 | 5'-CCGAAGGAGAAGCATATGAGAGCCG-3' | For cloning fragment used in pBIO2012 |
| RsphacuIBamREV1 | 5'-GGAGTGGGATCCGCGCTCTCTC-3' | For cloning fragment used in pBIO2012 |
| Spo11914_NdeF | 5’-ggaatccatATGTTCAATGCATTGGTGG-3’ | For cloning fragment used in pBIO2013 |
| HaloSphFOR1 | 5’-CGGCATGCGAGCGAAGCTAGAGCACAGC-3’ | For cloning fragment used in pBIO2014 |
| HaloXbaREV1 | 5’-CCTTTCTAGATTGATCAGGCGGTGTTCG-3’ | For cloning fragment used in pBIO2014 |
| M3AacuIPstFOR1 | 5'-CGCCGAGCTGCAGTACGCGGTCCGTCCC-3' | For cloning fragment used in pBIO2015 |
| M3AacuIEcoREV1 | 5'-GCGCCCGCAAGAATTCCCCCCAACATTCG-3' | For cloning fragment used in pBIO2015 |
| RlegacuINdeFOR1 | 5'-GACCGGGAGGCACCATATGACCGAGACC-3' | For cloning fragment used in pBIO2016 |
| RlegacuIBamREV1 | 5'-CGACAGTTCTGGATCCTGCC-3' | For cloning fragment used in pBIO2016 |
| SPO1913/14_XbaF | 5’-gcgtctagaggtcctgacgccgggtcgcac-3’ | For cloning fragment used in pBIO2019 |
| SPO1913/14_BamR | 5’-cgggatccgggcctcttgccgctcacttc-3’ | For cloning fragment used in pBIO2019 and pBIO2013 |
| M13F | 5'-CGCCAGGGTTTTCCCAGTCACGAC-3' | Sequencing |
| M13R | 5'-TCACACAGGAAACAGCTATGAC-3' | Sequencing |
| T7 20mer | 5'-TAATACGACTCACTATAGGG-3' | Sequencing |
| T7 terminator | 5'-TAATACGACTCACTATAGGG-3' | Sequencing |
| lacZ | 5'-GCCAGCTGGCGAAAGGGGGATGTGC-3' | Sequencing |
| pMP220 | 5'-GCTGCGCCCCTCGGCCGCGAACGGC-3' | Sequencing |
